# Patchouli Essential Oil and Its Derived Compounds Revealed Prebiotic-Like Effects in C57BL/6J Mice

**DOI:** 10.3389/fphar.2019.01229

**Published:** 2019-10-17

**Authors:** Waikit Leong, Guoxin Huang, Imran Khan, Wenrui Xia, Yucui Li, Yuhong Liu, Xiaoang Li, Ruixuan Han, Ziren Su, W. L. Wendy Hsiao

**Affiliations:** ^1^State Key Laboratory of Quality Research in Chinese Medicine, Macau University of Science and Technology, Macau, China; ^2^Guangdong Provincial Key Laboratory of New Chinese Medicinal Development and Research, Mathematical Engineering Academy of Chinese Medicine, Guangzhou University of Chinese Medicine, Guangzhou, China

**Keywords:** patchouli essential oil, patchouli alcohol, pogostone, β-patchoulene, gut microbiota

## Abstract

*Pogostemon cablin* (Blanco) Benth (PC) is a Chinese medicinal plant traditionally used for the treatment of gastrointestinal symptoms. To investigate the prebiotic effect of patchouli essential oil (PEO) and its derived compounds through the modulation of gut microbiota (GM). C57BL/6J mice were treated with the PEO and three active components of PEO, *i.e.* patchouli alcohol (PA), pogostone (PO) and β-patchoulene (β-PAE) for 15 consecutive days. Fecal samples and mucosa were collected for GM biomarkers studies. PEO, PA, PO, and β-PAE improve the gut epithelial barrier by altering the status of E-cadherin vs. N-cadherin expressions, and increasing the mucosal p-lysozyme and Muc 2. Moreover, the treatments also facilitate the polarization of M1 to M2 macrophage phenotypes, meanwhile, suppress the pro-inflammatory cytokines. Fecal microbial DNAs were analyzed and evaluated for GM composition by ERIC-PCR and 16S rRNA amplicon sequencing. The GM diversity was increased with the treated groups compared to the control. Further analysis showed that some known short chain fatty acids (SCFAs)-producing bacteria, *e.g. Anaerostipes butyraticus*, *Butytivibrio fibrisolvens*, *Clostridium jejuense*, *Eubacterium uniforme*, and *Lactobacillus lactis *were significantly enriched in the treated groups. In addition, the key SCFAs receptors, GPR 41, 43 and 109a, were significantly stimulated in the gut epithelial layer of the treated mice. By contract, the relative abundance of pathogens *Sutterlla* spp., *Fusobacterium mortiferum*, and *Helicobacter* spp. were distinctly reduced by the treatments with PEO and β-PAE. Our findings provide insightful information that the microbiota/host dynamic interaction may play a key role for the pharmacological activities of PEO, PA, PO, and β-PAE.

**Figure:**
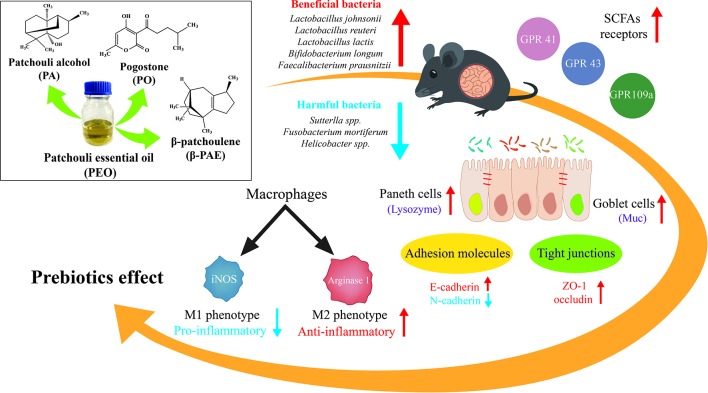
Graphical Abstract

## Introduction

Recent studies have revealed the critical role of gut microbiota (GM) in human health through a dynamic interaction between the host and the gut microbiota (Maslowski and Mackay, 2011; Marchesi et al., 2016). It is also known that diet is a key factor in the complex GM–host interplay. Recently, a group of dietary prebiotics, representing non-digestible fibers, polyphenols, and polysaccharides has raised great interest, as they exert beneficial effects by enhancing beneficial bacteria in the gut. More importantly, these bacteria convert the undigested food into functional metabolites(den Besten et al., 2013; Rowland et al., 2018). Short chain fatty acids (SCFAs) are the major bacterial metabolites with divert beneficial effects to the host energy metabolism and immune responses. Animal and epidemiological studies also reveal that SCFAs derived from the prebiotic uptake reduce the symptoms of various difficult diseases, such as autism, ulcerative colitis, Crohn’s disease, as well as cancer (Li et al., 2017; Pascal et al., 2017; Rea et al., 2018; Shen et al., 2018).

Traditional Chinese medicine (TCM) has compiled thousands of herbs and formula used for disease treatment as well as dietary supplements. Emerging evidences showed that the certain active constituencies from Chinese herbal medicines possess distinct prebiotic-like effects to which the inflammation and the cancer progress were alleviated (Chen et al., 2015b, Chen et al., 2015b; Huang et al., 2017). Thus, it has been speculated that Chinese herbal medicines might exert their medicinal functions through the modulation of GM and the gut epithelial environment to the benefit of the host (Jia et al., 2008; Li et al., 2009).

**Figure 1:**
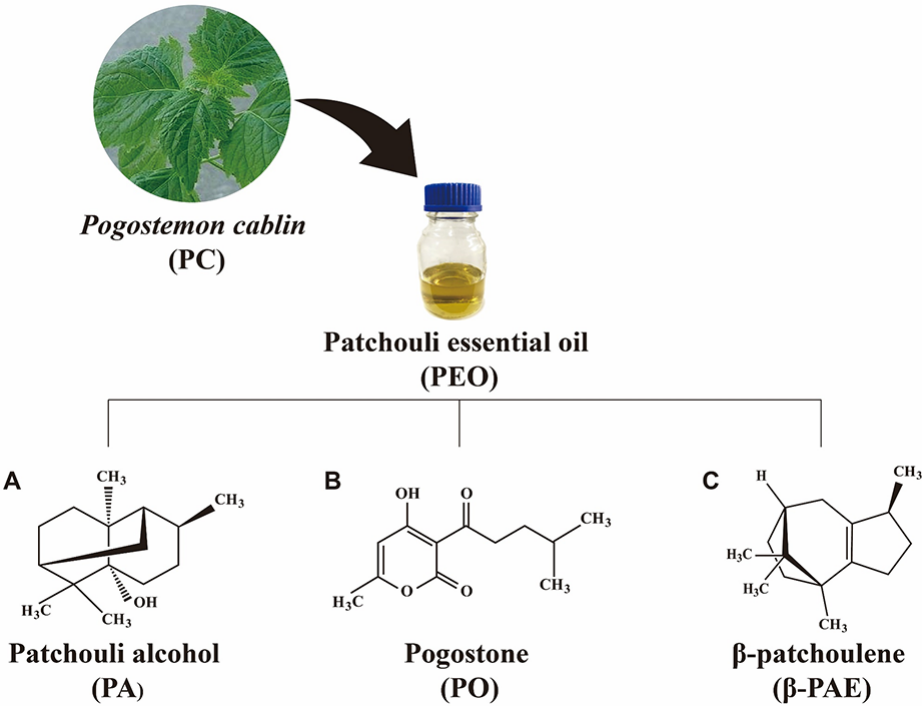
The chemical structure of **(A)** PA, **(B)** PO, and **(C)** β-PAE.

*Pogostemon cablin* (PC) is a medicinal herb commonly used for treating gastrointestinal symptoms, including colds, headaches, nausea, vomiting, abdominal pain, diarrhea, ulcerative colitis, dyspepsia, and poor appetite (Xian et al., 2011). Patchouli essential oil (PEO) extracted from the leaves of PC is the main active component and a well-known essential oil for various medicinal purposes. Patchouli alcohol (PA, C_15_H_26_O, **Figure 1A**) is a tricyclic sesquiterpene. It is the major component of PC and contribute to the pungent smell of the oil. Pogostone (PO, C_12_H_16_O_4_, **Figure 1B**) is a 2H-pyranone derived from PEO. β-patchoulene (β-PAE, C_15_H_24_, **Figure 1C**) that derived from PEO, is another representative hydrocarbon sesquiterpenoids of PEO. Accordingly, PA can convert to β-PAE by gastric juice with improved pharmacological outcome in animal (Liu et al., 2017). Both PA and PO have been designated as the chemical markers for the quality control of PC (Yang et al., 2016). PA, PO, and β-PAE compose of 39%, 8.9%, and 3.0% of PAO, respectively (van Beek and Joulain, 2018). These components of PC exhibited common as well as unique pharmaceutical activities (Li et al., 2014; Zhang et al., 2016a; Zhang et al., 2016b). For example, reports showed that PEO, PA, PO, and β-PAE possessed anti-inflammation, anti-oxidant, anti-cancer, immunomodulation in cellular and animal studies (Li et al., 2011; Liao et al., 2013; Jeong et al., 2013; Dechayont et al., 2017). Studies also showed that PEO, PA, and PO can suppress the growth of methicillin-resistant *Staphylococcus aureus*, *Escherichia coli*, and *Helicobacterium* both *in vitro* and *in vivo* experiments (Peng et al., 2014; Xie et al., 2016).

Based on the clinical observation that the primary pharmaceutical action of patchouli is on the gastrointestinal tract, we hypothesize that the medicinal properties of patchouli might be through the modulation of the GM composition. Thus, in this study, PEO, PA, PO, and β-PAE were evaluated for their impact on GM using ERIC-PCR and 16S rRNA gene sequencing. In addition, mucosal cytokines and the signaling molecules were investigated and compared between the treated and control healthy C57BL/6J mice.

## Materials and Methods

### Source of PEO, PA, PO, and β-PAE

PEO was purchased from Nanhai Zhongnan Co., Ltd. (Foshan, China; Lot 140801). PA, PO, and β-PAE were extracted from patchouli essential oil and analyzed according to the methods described in the previous reports (Li et al., 2012; Su et al., 2014; Zhang et al., 2016b). The purities of PA, PO and β-PAE are 99%, 98% and 98% respectively.

### Animals and Treatments

The C57BL/6J mice (6 weeks age) were purchased from the Chinese University, Hong Kong. All the mice were kept on a 12-h light and dark cycle under regulated temperature (22 ± 2°C) and humidity (50 ± 10%) with free access to food and water. Mice were fed with PicoLab® Rodent Diet 205053 (LabDiet, USA). The procedures were approved by the University Ethics Review Committee of the Macau University of Science and Technology. A total of 25 male C57BL/6J mice were randomly divided into five groups and gavage with non-toxic doses of PEO (40 mg/kg), and three pure compounds, PA (20 mg/kg), PO (20 mg/kg), β-PAE (20 mg/kg), and vehicle 0.5% carboxymethyl cellulose (CMC) and 1% dimethyl sulfoxide (DMSO) to control group for 15 consecutive days, and pentobarbital sodium was used for euthanasia of the mice. Non-toxic dosages were chosen for each test compound based on the previous reports (Li et al., 2011; Chen et al., 2015a).

### Genomic DNA Extraction From Fecal Samples

**Figure 2:**
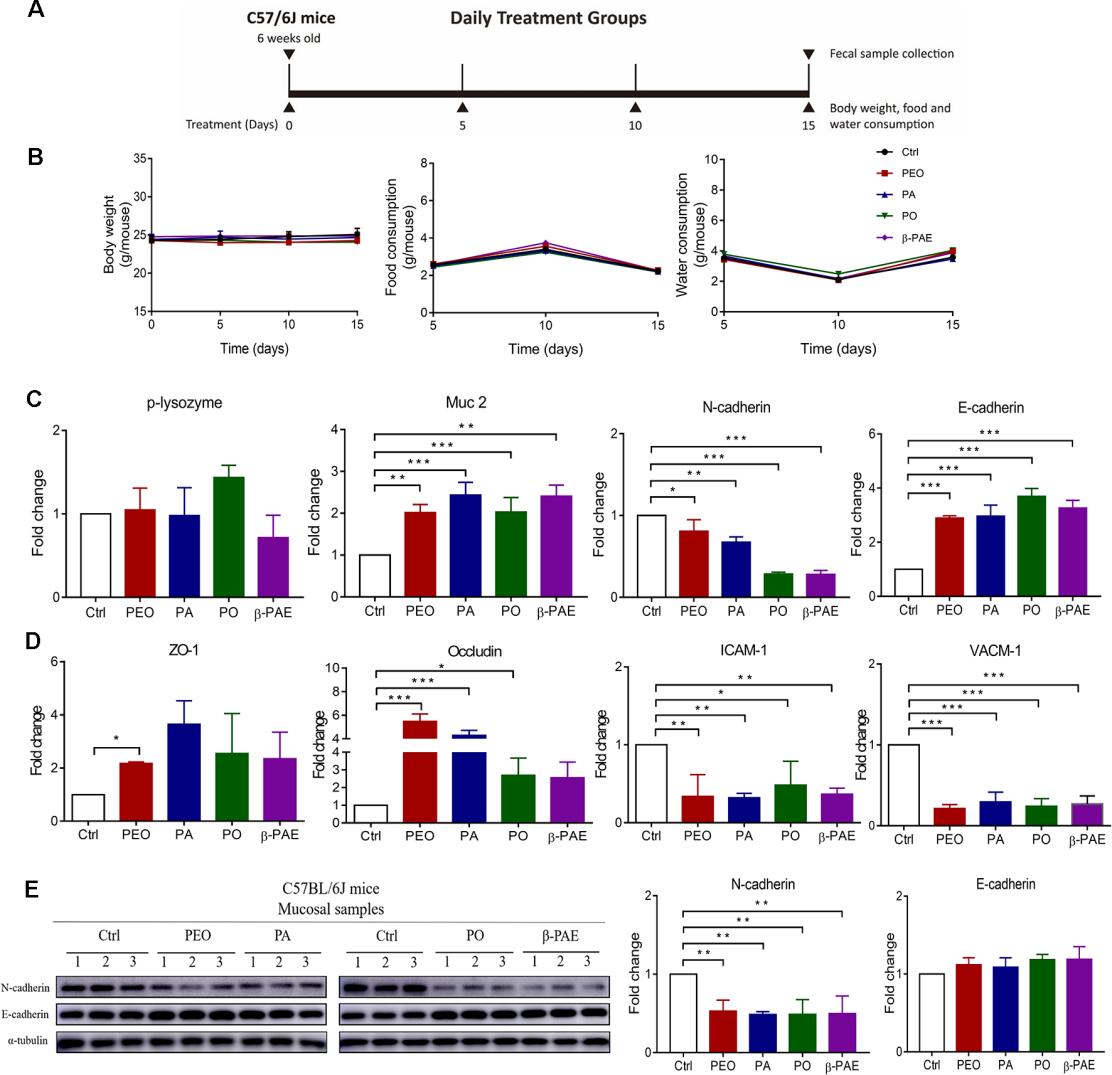
PEO, PA, PO and β-PAE differentially improved the gut epithelial barrier. **(A)** The treatment schemes. **(B)** The profiles of body weight, diet and water consumption (n = 5). **(C)** RNA expressions of p-lysozyme, Muc 2, N-cadherin and E-cadherin assessed by qRT-PCR. **(D)** RNA expressions of molecules of tight junctions and adhesion junctions. **(E)** Western blotting analysis for N-cadherin and E-cadherin in mucosa of the treated and the control groups. Full length of the 496 western blots is shown in **Supplementary Figure 3** Data is presented as the mean ± SEM. Statistical analysis was performed with one-way ANOVA. *p ≤ 0.05: **p ≤ 0.01 (n = 3); ***p ≤ 0.001 (n=3).

Animal fecal samples were collected from each individual mouse at D0, D5, D10 and D15, and directly stored at −80 °C for DNA extraction later. The treatment scheme was shown in **Figure 2A**. QIAamp DNA Stool Mini Kit (QIAGEN) was used to extract genomic DNA following the manufacturer’s manual and kept at −20 °C for later experiments.

### Enterobacterial Repetitive Intergenic Consensus (ERIC)-PCR

Fecal DNA samples were extracted to analyze for ERIC regions with a pair of ERIC primers sequences: ERIC 1 (5′-ATGTAAGCTCCTGGGGATTCAC-3′) and ERIC 2 (5′-AAGTAAGTGACTGGGGTGAGCG-3′). PCR conditions were carried out as previously described (Chen et al., 2015c). ERIC-PCR products were loaded in 2% agarose gel and ran at 100 v for 40 min in 1× TAE. The bands patterns were visualized with using the image Lab 5.2 system (Bio-Rad) and digitized for microbial clustering analysis using SIMCA-P 14.1 tool (Umetrics, Umea, Sweden) with confidence level 95% (p < 0.05).

### Quantitative Real Time Polymerase Chain Reaction (QRT-PCR) Analysis

qRT-PCR was performed according to our previous study (Fodde et al., 1994). The total RNA from the intestinal mucosal samples were isolated with RNeasy Mini Kit (QIAGEN, Hilden, Germen) according to the manufacturer’s protocol. The qRT-PCR working condition was followed to our previous study and performed using Applied Biosystems ViiA™ 7 PCR system (Carlsbad, CA, USA) (Chen et al., 2016). The 2−ΔΔCt method was applied to calculate the fold change of relative gene expression. ΔΔCt = (Ct treatment_target gene − Ct treatment_reference gene) − (Ct control_target gene − Ct control_reference gene). β-actin acted as the internal control for the relative quantification of target genes. The sequences of primers are all listed in **Supplementary Table 4**.

### Western Blot Analysis

Western blot analysis was performed with the standard method on the mucosal protein lysates from individual experimental mice. Primary antibodies including specific antibodies against N-Cadherin, E-Cadherin (1:1000, Cell Signaling Technology, USA) and anti-α-tubulin (1:1000, Santa Cruz Biotechnology, USA). Secondary antibody polyclonal goat anti-rabbit immunoglobulins/HRP (1:5000, Agilent Dako, Santa Clara). The detection was done using chemiluminescence (Luminol Reagent, Santa Cruz) and viewed on the Amersham Imager 600 (GE Healthcare, US). The relative protein bands were analyzed with ImageJ software (MD, USA). α-tubulin was used as an internal control.

### 16S rRNA Gene Sequencing

Illumina MiSeq (Illumina, San Diego) was used to sequence the fecal DNA samples, targeting the V3–V4 region with barcoded 515F and 806R universal primers. The detailed sequencing processes were described in the previous study (Dowd et al., 2008; Khan et al., 2018).

### Statistical Analysis

SPSS version 22 and GraphPad prism 7 were used for statistical analysis. Bacterial taxa data normality was ascertained with Kolmogorov–Smirnov D test. One-way ANOVA (for parametric data) and Kruskal–Wallis tests (for non-normal data) were performed to observe signiﬁcantly different bacterial taxa among the groups. Linear discriminant analysis effect size (LEfSe) was used for determining biologically and statically consistent changes in the GM composition. Alpha-diversity values were set at 0.05 whereas the threshold on the logarithmic score of linear discriminant analysis was ≥ 2.0 (Segata et al., 2011; Chumpitazi et al., 2015). Partial least squares discriminant analysis (PLS-DA) was performed to visualize the changes of microbial communities before and after treatments (SIMCA-P 14.0, Umetrics, Umea, Sweden) for which the confidence level was set at 95% (p < 0.05). Weight changes, diet and water consumption data was statistically analyzed using one-way ANOVA and Dunnett’s *post hoc* test using GraphPad Prism version 7.0 (GraphPad Software, San Diego, CA, USA).

## Results

### PEO, PA, PO, and β-PAE Improved the Gut Epithelial Barrier

Treatments with PEO, PA, PO, and β-PAE showed no significant differences in body weight, food and water consumptions between the treated and untreated groups (**Figure 2B**). *Muc 2* and *p-lysozyme* are two important genes encoded mucin and lysozyme respectively. Mucin secreted by the goblet cells and lysozyme secreted by Paneth cells protect the epithelial lining from the intrusion of pathogen. We observed that all four groups significantly up-regulated the *Muc 2* expression (later). Adhesion molecules and tight junctions are two important proteins that associate with the health of gut epithelial barrier. E-cadherin is a key adhesion molecule. Switching from E-cadherin to N-cadherin is associated with the poor prognosis of colonic cancer. In this study, all herbal-treatments downregulated the N-cadherin and upregulated E-cadherin in mice (**Figures 2C, E**). Moreover, we found that zona occluden-1 (ZO-1) and occludin tight junction molecules were significantly increased in the treated groups (**Figure 2D**). On the other hand, vascular cell adhesion molecule 1 (VCAM-1) and intercellular adhesion molecule 1 (ICAM-1) involved in inflammatory response were prominently down-regulated in the treated groups (**Figure 2D**).

### The Herbal Treatments Facilitated the Switching of M1 to M2 Phenotypes, as Well as Reinstated the Expressions of Inflammatory Cytokines

**Figure 3:**
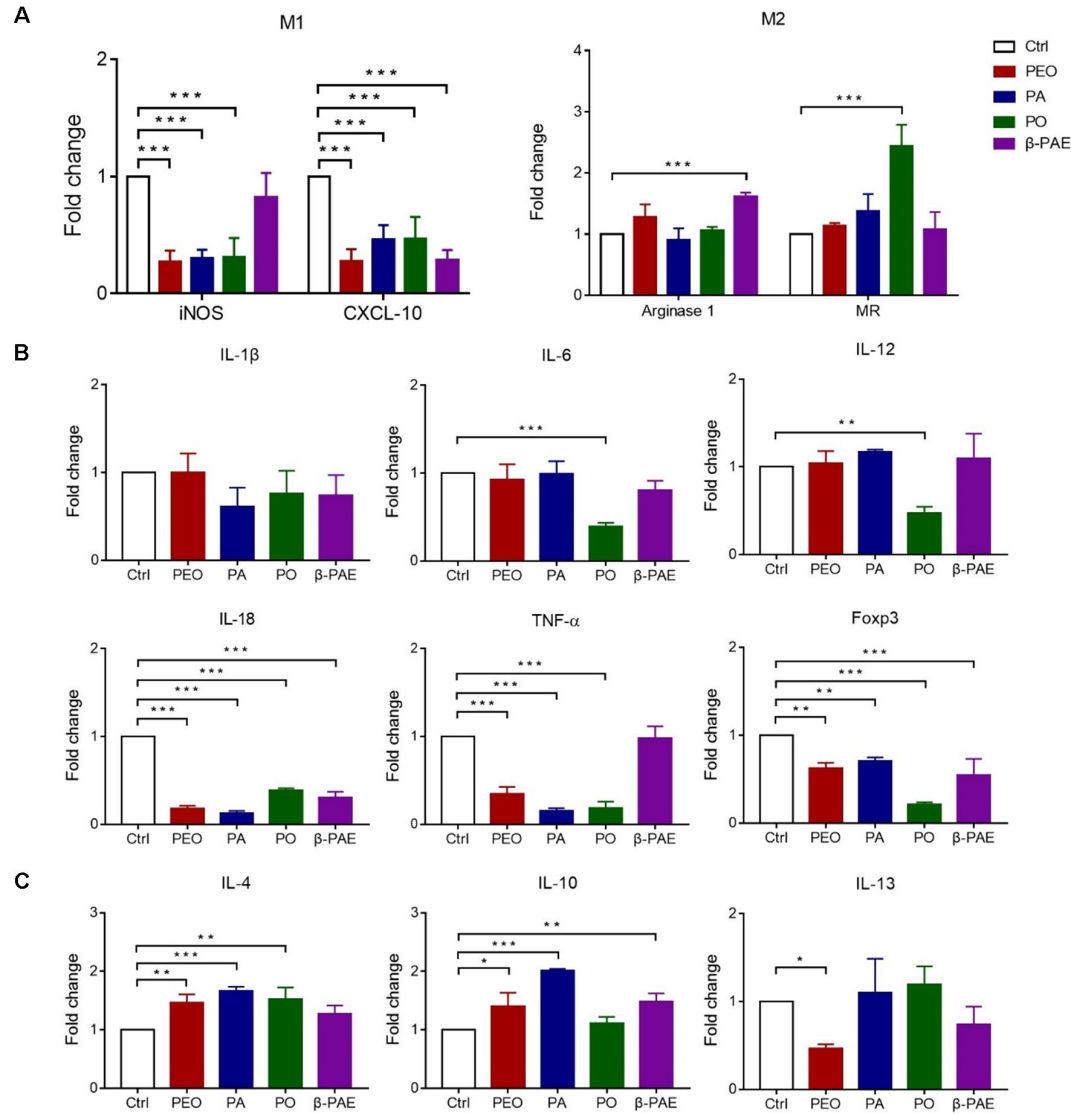
Effects of PEO, PA, PO and β-PAE on M1 and M2 macrophage phenotypes. **(A)** Relative RNA expressions of M1 and M2 macrophage markers by qRT-PCR. **(B)** and **(C)** The relative RNA expressions of pro/anti-inflammatory cytokines respectively. Data is presented as the mean ± SD, (n = 3). Statistical analysis was performed with one-way ANOVA. ^∗^p ≤ 0.05; ^∗∗^p ≤ 0.01; ***p ≤ 0.001.

It was well known that GM can significantly change the gut microenvironment, *e.g.* the intestinal immune system and the inflammation state. M1 and M2 macrophages are two macrophages that associated with gut immune system and inflammation. Here, we show the reduced expressions of M1 macrophage markers (iNOS and CXCL 10) in all treated groups by qRT-PCR detection. Meanwhile, arginase 1 and MR, the M2 markers were significantly increased in PO and β-PAE groups (**Figure 3A**). Moreover, the pro-inflammatory cytokines, IL-18, TNF-α and Foxp3 were distinctly down-regulated, while anti-inflammatory cytokines, IL-4, IL-10 and IL-13 were up-regulated in all treated mice compared to the control group (**Figures 3B, C**).

### Herbal Treatments Altered the Composition and Diversity of GM

**Figure 4:**
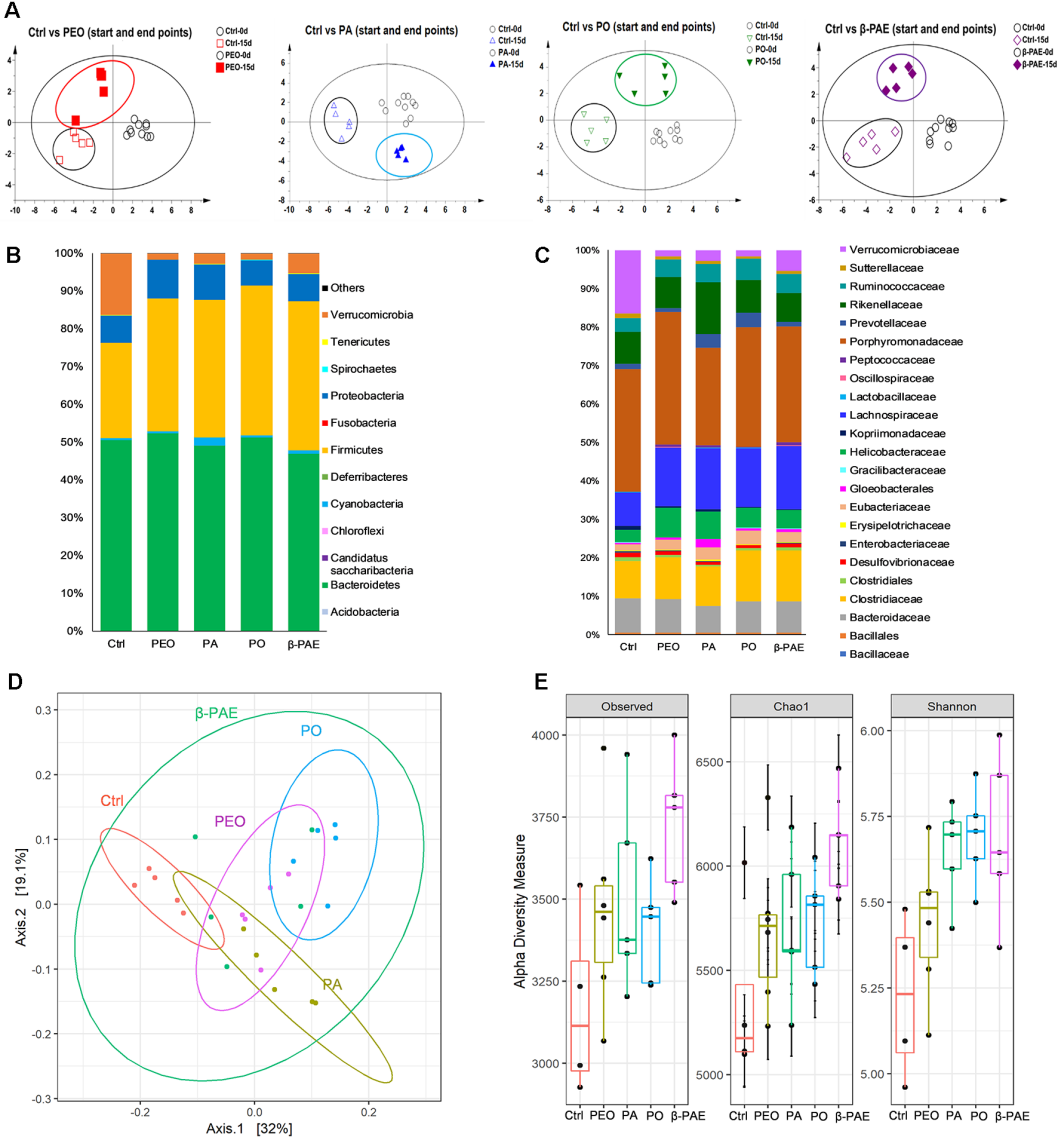
PEO, PA, PO and β-PAE changed the composition and diversity of GM. **(A)** PLS-DA plots of the ERIC-PCR results (n = 5). **(B)** and **(C)** Average relative abundance of the dominant bacterial phyla and family. **(D)** PCoA plots of GM profiles of the experimental mice using the weighted UniFrac matrix. **(E)** α-diversity analysis for the GM composition.

DNAs were isolated from D0 and D15 fecal samples. ERIC-PCR was used to analyze the GM composition. An average of 20 bands were generated in each sample, then the bands were digitized by Image Lab software (version 3.0) and analyzed with PLS-DA. PLS-DA results showed that clear separation of between the treated and control groups at D0 and D15 (**Figure 4A**). Meanwhile, DNAs from D15 fecal samples were further analyzed using 16S amplicon sequencing on MiSeq sequencer. More than 15 phyla and 25 families were detected. When compared with the control group, the relative abundance of phylum Firmicutes was increased, while the Verrucomicrobia was decreased in all treated groups. In family level, the abundance of Eubacteriaceae and Lachnospiraceae were both increased, however, Verrucomicrobia was decreased in all treated groups (**Figures 4B, C**). PCoA results displayed the significant clustering between the treated and control groups (**Figure 4D**). α-diversity analysis showed that the Observed, Chao 1, and Shannon indexes of all treated groups were increased compared with the control (**Figure 4E**).

### PA, PO and β-PAE Markedly Increased the Relative Abundance of Lactic Acid Producing Bacteria

**Figure 5:**
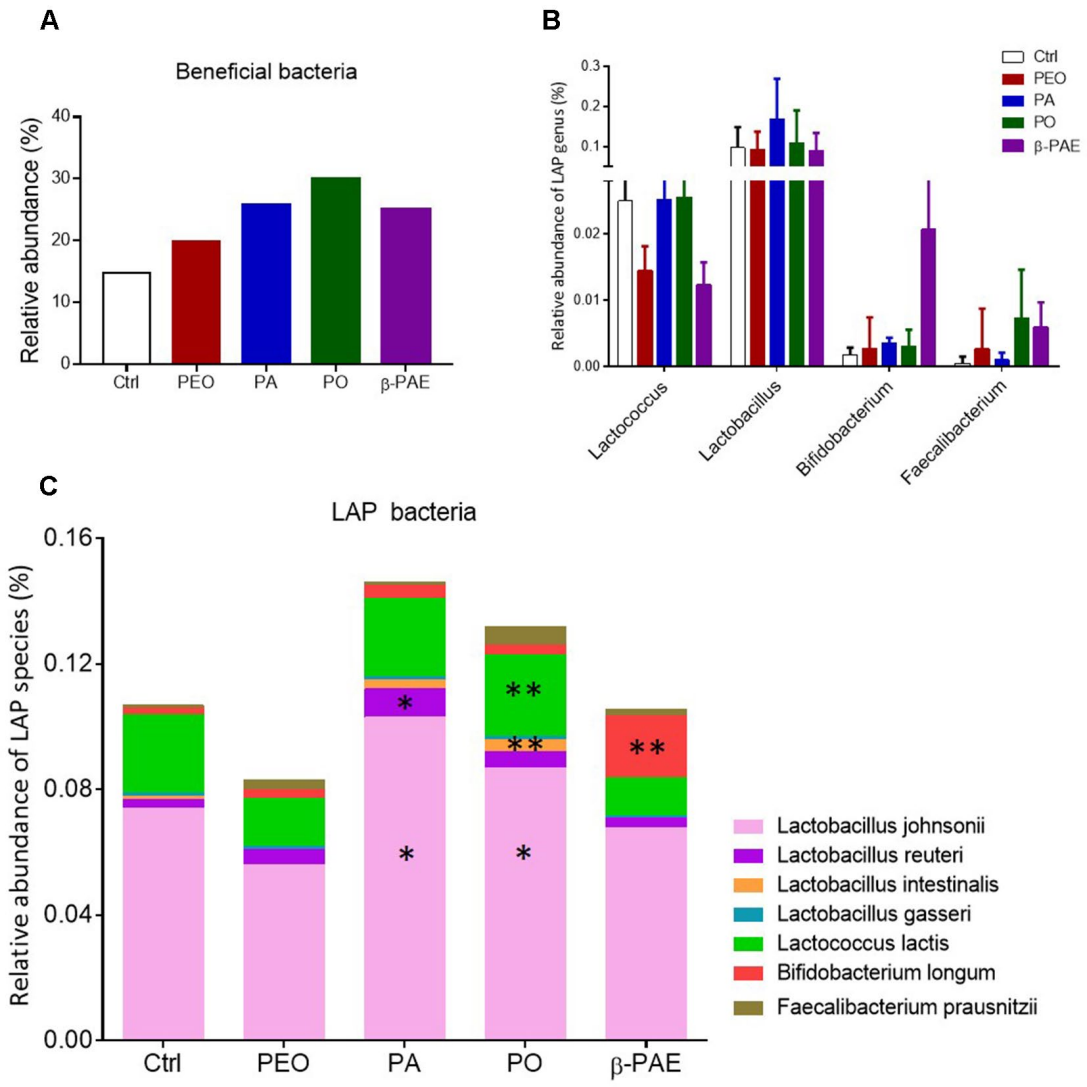
PA and PO markedly increased the relative abundance of LAP bacteria. **(A)** The relative abundance of beneficial bacteria in all treated groups. **(B)** The relative abundance of LAP bacteria in genera taxon. **(C)** The relative abundance of LAP bacteria in species taxon. ^∗^p ≤ 0.05; ^∗∗^p ≤ 0.01.

We compiled the known beneficial bacteria from the control and treatment groups based on the 16S sequencing data (**Supplementary Table 2**). An obvious boost of the over-all relative abundance of beneficial bacteria in the mice treated with PEO, PA, PO, and β-PAE (**Figure 5A**). Among the beneficial bacteria, the lactic acid producing (LAP) bacteria were markedly increased in the PA, PO, and β-PAE groups (**Figures 5B, C** and **Supplementary Table 3**). The genera *Lactobacillus*, and *Faecalibacterium* were increased with PA and PO treatments, while the *Bifidobacterium* were prominently enhanced in mice treated with β-PAE. At the species level, we found that the key LAP bacteria, such as *Lactobacillus johnsoni*, *Lactobacillus reuteri*, *Lactococcus lactis*, and *Faecalibacterium prausnitzii* were distinctly increased in the PA and PO groups. On the other hand, β-PAE treatment caused about 10 folds increase of *B. longum* (**Figures 5B, C**). LAP bacteria produce lactic acid to maintain the balance of intestinal pH. Reports also showed that LAP bacteria can improve host digestion and intestinal lining (Vigsnaes et al., 2013).

### The Herbal Treatments Differentially Enhanced the Abundance of Scfas Producing Bacteria and Activated the Scfas-Sensing Receptors in the Host Epithelial Barrier

**Figure 6:**
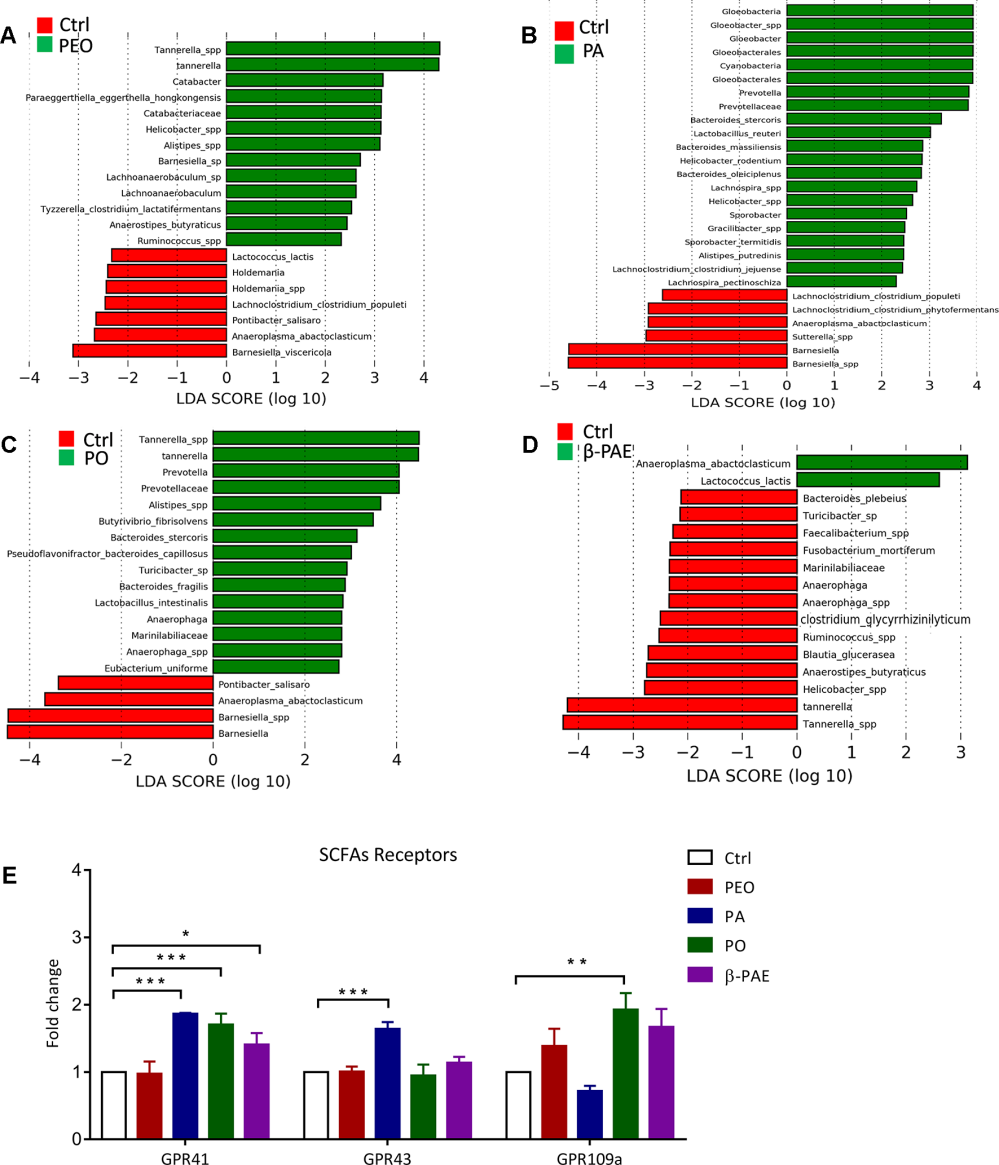
PEO, PA, PO, and β-PAE enriched the relative abundance of SCFAs-producing bacteria and affected its down molecular cascades. **(A)**, **(B)**, **(C)**, **(D)** LDA score for the untreated group versus treated groups. **(E)** The fold change of the mRNA expression of SCFAs receptors (GPR41, 43 and 109a). Data is presented as the mean ± SD. Statistical analysis was performed with one-way ANOVA. *p ≤ 0.05; **p ≤ 0.01; ***p ≤ 0.001.

SCFA is an important bacterial metabolite and exerts various bioactivities through the G-protein-coupled receptors residing in the gut epithelial layer. LEfSe analysis and LDA score showed that certain SCFAs producing bacteria, *e.g. Anaerostipes butyraticus*, *Clostridium lactatifermentans* in PEO group, *Prevotella* spp., *Clostridium jejuense*, and *Clostridium populeti* in PA group, *Eubacterium uniforme*, *Lactobacillus intestinalis* and *Butytivibrio fibrisolvens* in PO group, *Lactococcus lactis* in β-PAE group, were all significantly increased (**Figures 6A–D** and **Supplementary Figures 2A–D**). In order to impact of these bacteria to the host physiology, we tested the mRNA expressions of three main SCFAs receptors—GPR 41, 43 and 109a using qRT-PCR, and showed that all three receptors were significantly increased in all treated groups (**Figure 6E**). Meanwhile, we also noticed that certain harmful bacteria, such as *Sutterlla* spp., *Fusobacterium mortiferum*, and *Helicobacter* spp. associated with gastrointestinal inflammation and cancer, were significantly reduced in PEO and β-PAE groups (**Figures 6A, D** and **Supplementary Figures 2A–D**).

## Discussion

Patchouli is a traditional Chinese medicine for the treatment of gastrointestinal symptoms. PEO and its derived compounds PA, PO, and β-PAE are the main active ingredients of patchouli that possess anti-inflammation, anti-oxidant, anti-microbial, anti-allergy, and anti-cancer effects based on the cellular and animal studies (Li et al., 2011; He et al., 2013; Jeong et al., 2013; Dechayont et al., 2017). It is our interest to investigate whether the commensal GM plays a role in the health benefits of patchouli. In this study, we revealed several novel functions of the herbs taking place in the gut epithelial microenvironment. First of all, we observed the great improvement of the gut barriers of the treated mice, evidenced by the increased expressions of *p-lysozyme* and *Muc 2*, positively adjusting the E-cadherin/N-cadherin ratio. Down-regulation of E-cadherin and up-regulation of N-cadherin was usually detected in patients with colitis, Crohn’s disease, especially with colorectal cancer (Libusova et al., 2010; Schneider et al., 2010). Tight junction proteins play an important role in the structure and physiology of the gut epithelial barrier (Zeisel et al., 2019). In the treated mice, we detected marked enhancement of both cytoplasmic ZO-1 and transmembrane protein occludin. Both ZO-1 and the occludin are the major parts of the tight junction complex. Disruption of tight junctions integrity would increase the gut permeability and inflammation, and might lead to GI cancer (Park et al., 2015; Zeisel et al., 2019). On the other hand, VCAM-1 and ICAM-1 were prominently down-regulated in the treated groups. VCAM and ICAM are mainly expressed in the endothelial cells and responsible to recruit the leucocyte to activate the inflammatory response. Elevation of VCAM is known to contribute to cancer progression and metastasis (Schlesinger and Bendas, 2015).

Excessive inflammation could cause destruction of the gut epithelial barrier. In addition to safe guard the gut barrier, PEO, PA, PO, and β-PAE treatments also provided an anti-inflammatory gut microenvironment to the treated mice. Evidence included the shifting the pro-inflammatory M1 macrophages to the anti-inflammatory M2 macrophages based on the expressions of M1 biomarkers (iNOS and CXCL 10) and M2 macrophage biomarkers (arginase 1) (Luzina et al., 2012; Novak and Koh, 2013; Kühl et al., 2015). Moreover, we also quantified the pro-inflammatory and anti-inflammatory cytokines expressions in the mucosa of the mice. And the results showed that pro-inflammatory cytokines, *e.g.* IL-1β, IL-18, TNF-α, and Foxp3 were obviously reduced in the treated groups. This result echo other reports in which the expression of LPS-induced pro-inflammatory cytokines TNF-α, IL-1β, iNOS, and IL-6 were inhibited by the treatments with PA and PO (Jichlinski et al., 2003; Sun et al., 2016). We also showed that PEO, PA, PO, and β-PAE effectively up-regulated the anti-inflammatory cytokines IL-4 and IL-10.

The changes of the GM composition upon the herbal treatments were clearly displayed in the PLS-DA and PCoA charts (**Figure 4**) The most important effect of these herbal compounds is the enhancement of SCFA-producing bacteria and the activation of the SCFA-sensing GPCRs. SCFAs are the main energy source to the colonic cells and stimulate the growth of the gut epithelial cells (Cummings, 1981; Liu et al., 2016). Recent reports also demonstrated that SCFAs, especially butyrate possess immunomodulation, anti-inflammation, apoptosis induction, anti-cancer *via* directly or indirectly activated SCFAs receptors (GPRs 41, 43 and 109a). GPRs serve as the contact points between the GM and the host. The binding then trigger series of signaling cascades in GI. Report showed that GPR43-deficient mice displayed accelerated inflammation in the colitis, arthritis, and asthma mouse models. Study also showed that germ-free wild-type mice failed to convert fiber to SCFAs and showed worsened inflammatory conditions (Maslowski et al., 2009). Another report suggested that activation of GPR109a induced expressions of anti-inflammatory effector molecules and prevented colitis and colon carcinogenesis in animal studies (Singh et al., 2014; Rooks and Garrett, 2016).

In addition to the enhancement of SCFAs producing bacteria in the gut, we found that PA and PO post strong effect on the abundance of the LAP bacteria, especially for *L. johnsonii*, *L. reuteri*, *L. lactis*, *B. longum*, and *F. prausnitzii*. Recent reports showed that LAP bacteria colonized host mucosa can improve intestinal lining, nutrient absorption, and reduce symptoms of lactose intolerance and other food allergies (Mayo et al., 2008; Pfefferle et al., 2013). It is worthy mentioned that herbal treatments also significantly decrease the relative abundance of pathogens, including, *Sutterlla* spp., *F. mortiferum*, and *Helicobacter* spp. The high abundance of *Sutterlla* spp. has been detected in patients with autism and inflammatory bowel disease (Mukhopadhya et al., 2011; Wang et al., 2013). *F. mortiferum* and *Helicobacter* spp. have been clearly indicated as the gastrointestinal inflammation/cancer-driver in clinical studies (Cover and Blaser, 2009; Kostic et al., 2012).

## Conclusion

In summary, our study demonstrated that PEO, PA, PO, and β-PAE possess significant prebiotic-likes effect in the C57BL/6J mouse model. The herbal treatments improved the gut epithelial barrier by reinstating the expressions of E-cadherin and N-cadherin, up-regulating *p-lysozyme* and *Muc 2* genes expression, and suppressing the pro-inflammatory cytokine expressions. 16S sequencing data showed that the herbal treatments positively modulated the GM composition in which, the relative abundance of SCFA-producing bacteria and LAP bacteria were enhanced while certain pathogenic bacteria were reduced. Importantly, the treatments also significantly stimulated the expressions of the host’s GPRs 41, 43 and 109a that play critical roles in gut homeostasis. Our study suggests that the microbiota/host dynamic interaction might account, at least in part, for the pharmacological activities PEO and its derived constituents.

## Data Availability Statement

The data has been submitted to SRA NCBI under the project ID: PRJNA542252.

## Ethics Statement

Full name of the ethics committee: University Ethics Review Committee of the Macau University of Science and Technology The C57BL/6J mice (6 weeks age) were purchased from the Chinese University, Hong Kong. All the mice were kept on a 12h-hlight and dark cycle under regulated temperature (22 ± 2°C) and humidity (50 ± 10%) with free access to food and water. Mice were fed with PicoLab® Rodent Diet 205053 (LabDiet, USA). Pentobarbital sodium was used for euthanasia of the mice.

## Author Contributions

WL performed animal experiments, fecal samples collection, Eric-PCR, qPCR, data assembly and wrote part of paper. GH performed animal experiments and wrote the paper. IK performed data analysis and generated the figures. XL, WX and RH performed animal experiments. YCL, YHL, and ZS extracted medicinal herb. The supervision of WH designed research project, interpreted and discussed the results, revised and final approved the manuscript.

## Funding

This study was funded by The Science and Technology Development Fund, Macau SAR (File No. FDCT130-2016-A3 & 0054-2018-A2) to WH.

## Conflict of Interest

The authors declare that the research was conducted in the absence of any commercial or financial relationships that could be construed as a potential conflict of interest.

## Abbreviations

CMC, Carboxymethyl cellulose; DMSO, Dimethyl sulfoxide; ERIC, Enterobacterial Repetitive Intergenic Consensus; GM, Gut microbiota; ICAM-1, Intercellular adhesion molecule 1; LAP, Lactic acid producing; PA, Patchouli alcohol; PC, *Pogostemon cablin*; PEO, Patchouli essential oil; PLS-DA, Partial least squares discriminant analysis; PO, Pogostone; qRT-PCR, Quantitative real time polymerase chain reaction; SCFAs, Short chain fatty acids; TCM, Traditional Chinese medicine; VCAM-1, Vascular cell adhesion molecule 1; ZO-1, Zona occluden-1; β-PAE, β-patchoulene; LDA, Linear Discriminant Analysis.

## References

[B1] Chen H. Liao H. Liu Y. Zheng Y. Wu X. Su Z. (2015a). Protective effects of pogostone from Pogostemonis Herba against ethanol-induced gastric ulcer in rats. Fitoterapia. 100, 110–107 10.1016/j.fitote.2014.11.017 25481373

[B2] Chen L. Brar M. S. Leung F. C. C. Hsiao W. L. W. (2016). Triterpenoid herbal saponins enhance beneficial bacteria, decrease sulfate-reducing bacteria, modulate inflammatory intestinal microenvironment and exert cancer preventive effects in ApcMin/+ mice. Oncotarget 7, 31226–31242. 10.18632/oncotarget.8886 27121311 PMC5058752

[B3] Chen L. Tai W. C. S. Hsiao W. L. W. (2015b). Dietary saponins from four popular herbal tea exert prebiotic-like effects on gut microbiota in C57BL/6 mice. J. Funct. Foods 17, 892–902. 10.1016/j.jff.2015.06.050

[B4] Chen L. Tai W. C. S. S. Brar M. S. Leung F. C. C. C. Hsiao W. L. W. W. (2015c). Tumor grafting induces changes of gut microbiota in athymic nude mice in the presence and absence of medicinal Gynostemma saponins. PLoS One 10 (5), e0126807. 10.1371/journal.pone.0126807 25992551 PMC4439139

[B5] Chumpitazi B. P. Cope J. L. Hollister E. B. Tsai C. M. McMeans A. R. Luna R. A. (2015). Randomised clinical trial: gut microbiome biomarkers are associated with clinical response to a low FODMAP diet in children with the irritable bowel syndrome. Aliment. Pharmacol. Ther. 42, 418–427. 10.1111/apt.13286 26104013 PMC4514898

[B6] Cover T. L. Blaser M. J. (2009). Helicobacter pylori in Health and Disease. Gastroenterology 136 (6), 1863–1873. 10.1053/j.gastro.2009.01.073 19457415 PMC3644425

[B7] Cummings J. H. (1981). Short chain fatty acids in the human colon. Gut 22, 763–779. 10.1136/gut.22.9.763 7028579 PMC1419865

[B8] Dechayont B. Ruamdee P. Poonnaimuang S. Mokmued K. Chunthorng-Orn J. (2017). Antioxidant and Antimicrobial Activities of Pogostemon cablin (Blanco) Benth. J. Bot. 1–6. 10.1155/2017/8310275

[B9] den Besten G. van Eunen K. Groen A. K. Venema K. Reijngoud D.-J. Bakker B. M. (2013). The role of short-chain fatty acids in the interplay between diet, gut microbiota, and host energy metabolism. J. Lipid Res. 54 (9), 2325–2340. 10.1194/jlr.R036012 23821742 PMC3735932

[B10] Dowd S. E. Sun Y. Wolcott R. D. Domingo A. Carroll J. A. (2008). Bacterial Tag–Encoded FLX Amplicon Pyrosequencing (bTEFAP) for Microbiome Studies: Bacterial Diversity in the Ileum of Newly Weaned Salmonella -Infected Pigs. Foodborne Pathog. Dis. 5, 459–472. 10.1089/fpd.2008.0107 18713063

[B11] Fodde R. Edelmannt W. Yangt K. Van Leeuwen C. Carlsont C. Renault B. (1994). A targeted chain-termination mutation in the mouse Apc gene results in multiple intestinal tumors (gene targetlng/colorectal cancer/animal model for human disease). Genetics 91, 8969–8973. 10.1073/pnas.91.19.8969 PMC44728 8090754

[B12] He J. J. Chen H. M. Li C. W. Wu D. W. Wu X. L. Shi S. J. (2013). Experimental study on antinociceptive and anti-allergy effects of patchouli oil. J. Essent. Oil Res. 25, 488–496. 10.1080/10412905.2013.809319

[B13] Huang G. Khan I. Li X. Chen L. Leong W. Ho L. T. (2017). Ginsenosides Rb3 and Rd reduce polyps formation while reinstate the dysbiotic gut microbiota and the intestinal microenvironment in ApcMin/+ mice. Sci. Rep. 7, 12552. 10.1038/s41598-017-12644-5 28970547 PMC5624945

[B14] Liao J. B. Wu D. W. Peng S. Z. Xie J. H. Li Y. C. Su J. Y. (2013). Immunomodulatory potential of patchouli alcohol isolated from Pogostemon cablin (Blanco) Benth (Lamiaceae) in mice. Trop. J. Pharm. Res. 12, 559–565. 10.4314/tjpr.v12i4.18

[B15] Jeong J. B. Choi J. Lou Z. Jiang X. Lee S. H. (2013). Patchouli alcohol, an essential oil of Pogostemon cablin, exhibits anti-tumorigenic activity in human colorectal cancer cells. Int. Immunopharmacol. 16, 184–190. 10.1016/j.intimp.2013.04.006 23602914

[B16] Jia W. Li H. Zhao L. Nicholson J. K. (2008). Gut microbiota: A potential new territory for drug targeting. Nat. Rev. Drug Discov. 7 (2), 123–129. 10.1038/nrd2505 18239669

[B17] Jichlinski P. Guillou L. Karlsen S. Malmstrom P. Jocham D. Brennhovd B. (2003). Hexyl aminolevulinate fluorescence cystoscopy: a new diagnostic tool for the photodiagnosis of superficial bladder cancer - a multicenter study. J. Urol. 170, 226–229. 10.1097/01.ju.0000060782.52358.04 12796694

[B18] Khan I. Huang G. Li X. Leong W. Xia W. Hsiao W. L. W. (2018). Mushroom polysaccharides from Ganoderma lucidum and Poria cocos reveal prebiotic functions. J. Funct. Foods 41, 191–201. 10.1016/j.jff.2017.12.046

[B19] Kostic A. D. Gevers D. Pedamallu C. S. Michaud M. Duke F. Earl A. M. (2012). Genomic analysis identifies association of Fusobacterium with colorectal carcinoma. Genome Res. 22, 292–298. 10.1101/gr.126573.111 22009990 PMC3266036

[B20] Kühl A. A. Erben U. Kredel L. I. Siegmund B. (2015). Diversity of intestinal macrophages in inflammatory bowel diseases. Front. Immunol. 6, 1–7. 10.3389/fimmu.2015.00613 26697009 PMC4670857

[B21] Li H. Zhou M. Zhao A. Jia W. (2009). Traditional Chinese medicine: Balancing the gut ecosystem. Phyther. Res. 23 (9), 1332–1335. 10.1002/ptr.2590 19253310

[B22] Li Q. Han Y. Dy A. B. C. Hagerman R. J. (2017). The Gut Microbiota and Autism Spectrum Disorders. Front. Cell. Neurosci. 11, 120. 10.3389/fncel.2017.00120 28503135 PMC5408485

[B23] Li Y. C. Liang H. C. Chen H. M. Tan L. R. Yi Y. Y. Qin Z. (2012). Anti-Candida albicans activity and pharmacokinetics of pogostone isolated from Pogostemonis Herba. Phytomedicine 20 (1), 77-83. 10.1016/j.phymed.2012.08.008 23159370

[B24] Li Y. C. Xian Y. F. Ip S. P. Su Z. R. Su J. Y. He J. J. (2011). Anti-inflammatory activity of patchouli alcohol isolated from Pogostemonis Herba in animal models. Fitoterapia 82, 129+5–1301. 10.1016/j.fitote.2011.09.003 21958968

[B25] Li Y. Xian Y. Su Z. Ip S. Xie J. Liao J. (2014). Pogostone suppresses proin fl ammatory mediator production and protects against endotoxic shock in mice. J. Ethnopharmacol. 157, 212–221. 10.1016/j.jep.2014.09.023 25256685

[B26] Libusova L. Stemmler M. P. Hierholzer A. Schwarz H. Kemler R. (2010). N-cadherin can structurally substitute for E-cadherin during intestinal development but leads to polyp formation. Development 137, 2297–2305. 10.1242/dev.048488 20534673

[B27] Liu Y. Liang J. Wu J. Chen H. Zhang Z. Yang H. (2017). Transformation of patchouli alcohol to β-patchoulene by gastric juice: β-patchoulene is more effective in preventing ethanol-induced gastric injury. Sci. Rep. 7, 1–13. 10.1038/s41598-017-05996-5 28717228 PMC5514077

[B28] Liu Z. Chen Z. Guo H. He D. Zhao H. Wang Z. (2016). The modulatory effect of infusions of green tea, oolong tea, and black tea on gut microbiota in high-fat-induced obese mice. Food Funct. 7, 4869–4879. 10.1039/C6FO01439A 27812583

[B29] Luzina I. G. Keegan A. D. Heller N. M. Rook G. A. W. Shea-Donohue T. Atamas S. P. (2012). Regulation of inflammation by interleukin-4: a review of “alternatives.”. J. Leukoc. Biol. 92, 753–764. 10.1189/jlb.0412214 22782966 PMC3441310

[B30] Marchesi J. R. Adams D. H. Fava F. Hermes G. D. A. Hirschfield G. M. Hold G. (2016). The gut microbiota and host health: A new clinical frontier. Gut 65, 330–339. 10.1136/gutjnl-2015-309990 26338727 PMC4752653

[B31] Maslowski K. Vieira A. Ng A. Kranich J. Sierro F. Yu D. (2009). Regulation of inflammatory responses by gut microbiota and chemoattractan receptor GPR43. Nature 461, 1282–1286. 10.1038/nature08530 19865172 PMC3256734

[B32] Maslowski K. M. Mackay C. R. (2011). Diet, gut microbiota and immune responses. Nat. Immunol. 12, 5–9. 10.1038/ni0111-5 21169997

[B33] Mayo B. Sinderen D. Ventura M. (2008). Genome Analysis of Food Grade Lactic Acid-Producing Bacteria: From Basics to Applications. Curr. Genomics 9, 169–183. 10.2174/138920208784340731 19440514 PMC2679651

[B34] Mukhopadhya I. Hansen R. Nicholl C. E. Alhaidan Y. A. Thomson J. M. Berry S. H. (2011). A comprehensive evaluation of colonic mucosal isolates of sutterella wadsworthensis from inflammatory bowel disease. PLoS One 6 (10), e27076. 10.1371/journal.pone.0027076 22073125 PMC3205041

[B35] Novak M. L. Koh T. J. (2013). Macrophage phenotypes during tissue repair. J. Leukoc. Biol. 93, 875–881. 10.1189/jlb.1012512 23505314 PMC3656331

[B36] Park H. Y. Kunitake Y. Hirasaki N. Tanaka M. Matsui T. (2015). Theaflavins enhance intestinal barrier of Caco-2 Cell monolayers through the expression of AMP-activated protein kinase-mediated Occludin, Claudin-1, and ZO-1. Biosci. Biotechnol. Biochem. 79 (1), 130–137. 10.1080/09168451.2014.951027 25175351

[B37] Pascal V. Pozuelo M. Borruel N. Casellas F. Campos D. Santiago A. (2017). A microbial signature for Crohn’s disease. Gut 66, 813–822. 10.1136/gutjnl-2016-313235 28179361 PMC5531220

[B38] Peng F. Wan F. Xiong L. Peng C. Dai M. Chen J. P. (2014). In vitro and in vivo antibacterial activity of Pogostone. Chin. Med. J. (Engl.) 127, 4001–4005. 10.3760/cma.j.issn.0366-6999.20140494 25430439

[B39] Pfefferle P. I. Prescott S. L. Kopp M. (2013). Microbial influence on tolerance and opportunities for intervention with prebiotics/probiotics and bacterial lysates. J. Allergy Clin. Immunol. 131, 1453–63; quiz 1464. 10.1016/j.jaci.2013.03.020 23643095

[B40] Rea D. Coppola G. Palma G. Barbieri A. Luciano A. Del Prete P. (2018). Microbiota effects on cancer: from risks to therapies. Oncotarget 9, 17915–17927. 10.18632/oncotarget.24681 29707157 PMC5915165

[B41] Rooks M. G. Garrett W. S. (2016). Gut microbiota, metabolites and host immunity. Nat. Rev. Immunol. 16, 341–352. 10.1038/nri.2016.42 27231050 PMC5541232

[B42] Rowland I. Gibson G. Heinken A. Scott K. Swann J. Thiele I. (2018). Gut microbiota functions: metabolism of nutrients and other food components. Eur. J. Nutr. 57 (1), 1–24. 10.1007/s00394-017-1445-8 PMC5847071 28393285

[B43] Schlesinger M. Bendas G. (2015). Vascular cell adhesion molecule-1 (VCAM-1) - An increasing insight into its role in tumorigenicity and metastasis. Int. J. Cancer 136, 2504–2514. 10.1002/ijc.28927 24771582

[B44] Schneider M. R. Dahlhoff M. Horst D. Hirschi B. Trülzsch K. Müller-Höcker J. (2010). A key role for E-cadherin in intestinal homeostasis and paneth cell maturation. PLoS One 5 (12), e14325. 10.1371/journal.pone.0014325 21179475 PMC3001873

[B45] Segata N. Izard J. Waldron L. Gevers D. Miropolsky L. Garrett W. S. (2011). Metagenomic biomarker discovery and explanation. Genome Biol. 12, R60. 10.1186/gb-2011-12-6-r60 21702898 PMC3218848

[B46] Shen Z. H. Zhu C. X. Quan Y. S. Yang Z. Y. Wu S. Luo W. W. (2018). Relationship between intestinal microbiota and ulcerative colitis: Mechanisms and clinical application of probiotics and fecal microbiota transplantation. World J. Gastroenterol. 24, 5–14. 10.3748/wjg.v24.i1.5 29358877 PMC5757125

[B47] Singh N. Gurav A. Sivaprakasam S. Brady E. Padia R. Shi H. (2014). Activation of Gpr109a, receptor for niacin and the commensal metabolite butyrate, suppresses colonic inflammation and carcinogenesis. Immunity 40, 128–139. 10.1016/j.immuni.2013.12.007 24412617 PMC4305274

[B48] Su Z. Q. Wu X. L. Bao M. J. Li C. W. Kong S. Z. Su Z. R. (2014). Isolation of (-)-patchouli alcohol from patchouli oil by fractional distillation and crystallization. Trop. J. Pharm. Res. 13 (3), 359–363. 10.4314/tjpr.v13i3.7

[B49] Sun C. Y. Xu L. Q. Zhang Z. B. Chen C. H. Huang Y. Z. Su Z. Q. (2016). Protective effects of pogostone against LPS-induced acute lung injury in mice via regulation of Keap1-Nrf2/NF-κB signaling pathways. Int. Immunopharmacol. 32, 55–61. 10.1016/j.intimp.2016.01.007 26800098

[B50] van Beek T. A. Joulain D. (2018). The essential oil of patchouli, Pogostemon cablin: A review. Flavour Fragr. J. 33, 6–51. 10.1002/ffj.3418

[B51] Vigsnaes L. K. van den Abbeele P. Sulek K. Frandsen H. L. Steenholdt C. Brynskov J. (2013). Microbiotas from UC patients display altered metabolism and reduced ability of LAB to colonize mucus. Sci. Rep. 3, 1110. 10.1038/srep01110 23346367 PMC3552269

[B52] Wang L. Christophersen C. T. Sorich M. J. Gerber J. P. Angley M. T. Conlon M. A. (2013). Increased abundance of Sutterella spp. and Ruminococcus torques in feces of children with autism spectrum disorder. Mol. Autism. 4 (1), 42. 10.1186/2040-2392-4-42 24188502 PMC3828002

[B53] Xian Y. F. Li Y. C. Ip S. P. Lin Z. X. Lai X. P. Su Z. R. (2011). Anti-inflammatory effect of patchouli alcohol isolated from Pogostemonis Herba in LPS-stimulated RAW264.7 macrophages. Exp. Ther. Med. 2 (3), 545–550. 10.3892/etm.2011.233 22977538 PMC3440699

[B54] Xie J. Lin Z. Xian Y. Kong S. Lai Z. Ip S. (2016). (-)-Patchouli alcohol protects against Helicobacter pylori urease-induced apoptosis, oxidative stress and inflammatory response in human gastric epithelial cells. Int. Immunopharmacol. 35, 43–52. 10.1016/j.intimp.2016.02.022 27017292

[B55] Yang Y. Kong W. Feng H. Dou X. Zhao L. Xiao Q. (2016). Quantitative and fingerprinting analysis of Pogostemon cablin based on GC-FID combined with chemometrics. J. Pharm. Biomed. Anal. 121, 84–90. 10.1016/j.jpba.2016.01.012 26799976

[B56] Zeisel M. B. Dhawan P. Baumert T. F. (2019). Tight junction proteins in gastrointestinal and liver disease. Gut 68, 547–561. 10.1136/gutjnl-2018-316906 PMC6453741 30297438

[B57] Zhang R. Yan P. Li Y. Xiong L. Gong X. Peng C. (2016a). A pharmacokinetic study of patchouli alcohol after a single oral administration of patchouli alcohol or patchouli oil in rats. Eur. J. Drug Metab. Pharmacokinet. 41, 441–448. 10.1007/s13318-015-0272-7 25753831

[B58] Zhang Z. Chen X. Chen H. Wang L. Liang J. Luo D. (2016b). Anti-inflammatory activity of β-patchoulene isolated from patchouli oil in mice. Eur. J. Pharmacol. 781, 229–238. 10.1016/j.ejphar.2016.04.028 27090925

