# Supplementary material for: Patchouli Essential Oil and Its Derived Compounds Revealed Prebiotic-Like Effects in C57BL/6J Mice
Source: Front Pharmacol. 2019 Oct 17;10:1229. doi: 10.3389/fphar.2019.01229 (PMC6812344; doi:10.3389/fphar.2019.01229)
Supplement: Supplementary file 1 [file DataSheet_1.docx]

**Supplementary Materials**


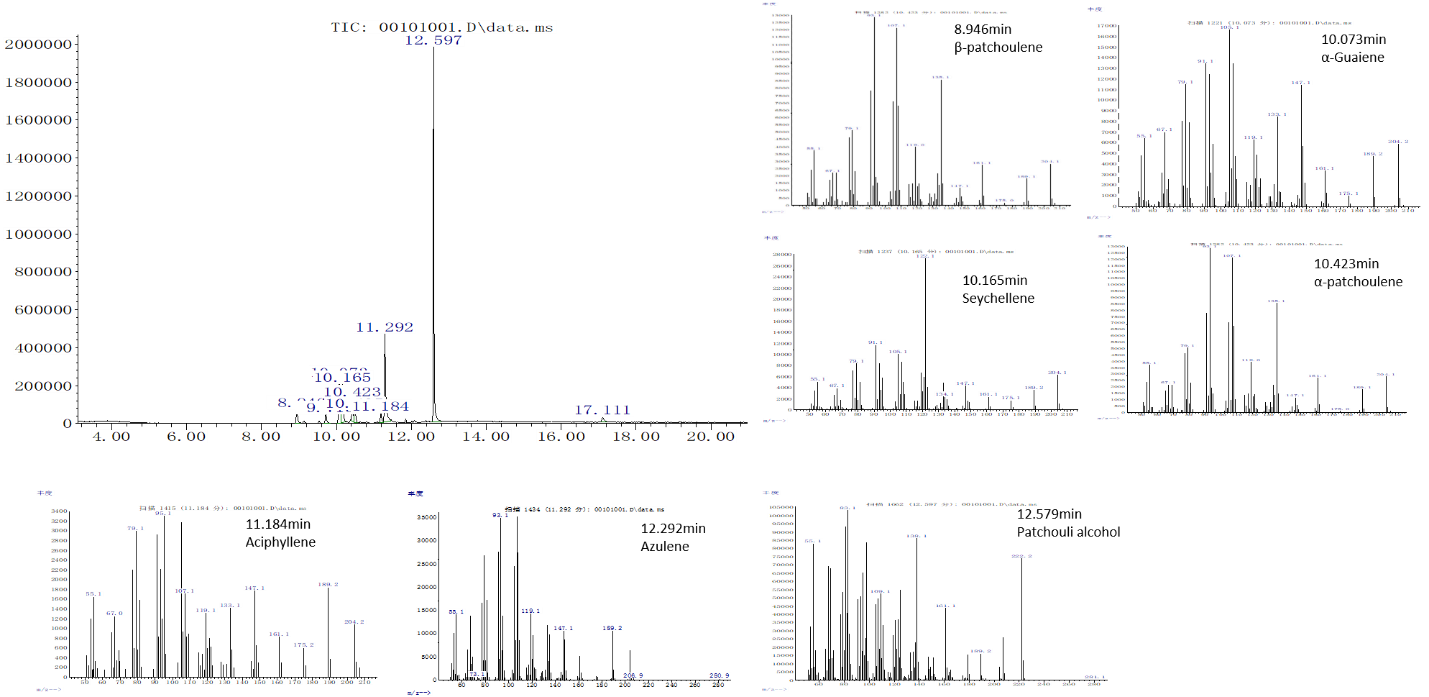


**Figure S1. The chemical profile of PEO using GC-MS.**

**Table S1. The relative abundance of these constitutions is descripted as follow:**

| **Name** | **Peak time/ min** | **Relative abundance/%** |
| --- | --- | --- |
| β-patchoulene | \| 8.946 \| \| --- \| | 3.673 |
| α-Guaiene | 10.073 | 11.348 |
| Seychellene | 10.165 | 9.068 |
| α-patchoulene | 10.423 | 4.733 |
| Aciphyllene | 11.184 | 2.381 |
| Azulene | \| 11.292 \| \| --- \| | 20.902 |
| Patchouli alcohol | \| 12.597 \| \| --- \| | 47.894 |

**Figure S2.** LefSe analysis for the untreated group versus treated groups.


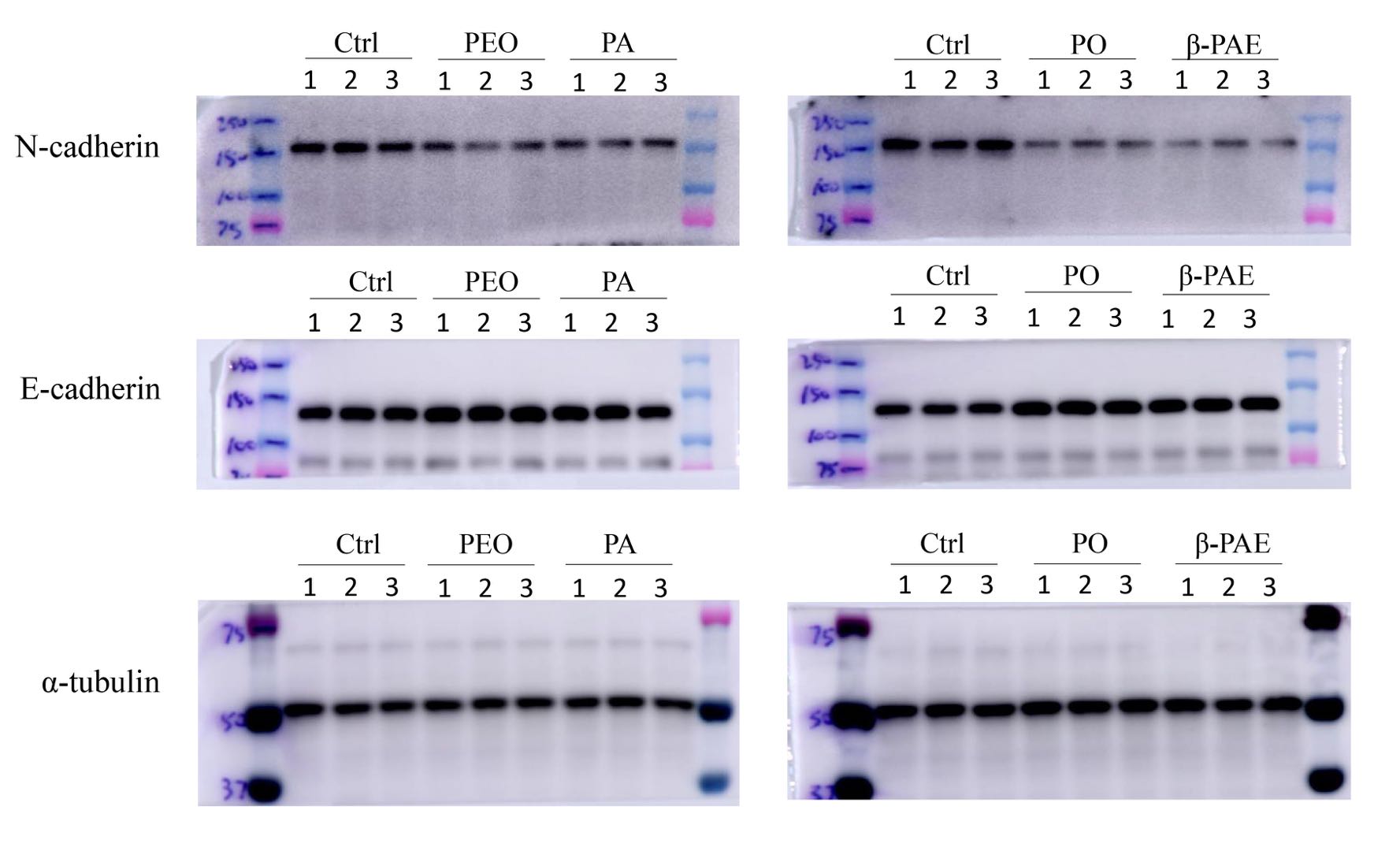


**Figure S3. The full** **length of Western Blotting.**

**Table S2. List of beneficial bacteria**

|  | Ctrl | PEO | | PA | | PO | | β-PAE | |
| --- | --- | --- | --- | --- | --- | --- | --- | --- | --- |
|  | Abundance | Abundance | Percentile change | Abundance | Percentile change | Abundance | Percentile change | Abundance | Percentile change |
| *Bacteroides xylanolyticus* | 0.415 | 0.830 | 100.103 | 1.466 | 253.262 | 1.780 | 328.927 | 1.080 | 160.186 |
| *Bifidobacterium longum* | 0.002 | 0.003 | 55.163 | 0.004 | 102.296 | 0.003 | 78.937 | 0.020 | 1055.792 |
| *Clostridium aminobutyricum* | 0.001 | 0.001 | -18.811 | 0.001 | -44.708 | 0.001 | -3.801 | 0.002 | 82.712 |
| *Clostridium sp.* | 5.688 | 6.229 | 9.500 | 6.223 | 9.395 | 8.636 | 51.809 | 8.223 | 44.553 |
| *Clostridium spp.* | 2.637 | 2.319 | -12.046 | 3.072 | 16.495 | 2.398 | -9.066 | 2.567 | -2.651 |
| *Clostridium sulfatireducens* | 1.147 | 1.930 | 68.232 | 0.554 | -51.710 | 1.771 | 54.424 | 2.001 | 74.434 |
| *Eubacterium cellulosolvens* | 0.092 | 0.212 | 129.675 | 0.146 | 58.276 | 0.360 | 291.046 | 0.455 | 394.180 |
| *Eubacterium spp.* | 1.230 | 1.845 | 49.941 | 2.406 | 95.573 | 2.634 | 114.121 | 1.638 | 33.132 |
| *Faecalibacterium prausnitzii* | 0.0009 | 0.003 | 464.505 | 0.001 | 120.384 | 0.006 | 1124.009 | 0.002 | 260.174 |
| *Lachnoclostridium clostridium aldenense* | 0.482 | 0.767 | 59.101 | 0.933 | 93.545 | 2.102 | 336.014 | 1.313 | 172.373 |
| *Lachnoclostridium clostridium aminovalericum* | 0.002 | 0.011 | 373.677 | 0.020 | 806.287 | 0.026 | 1076.326 | 0.012 | 447.430 |
| *Lachnoclostridium clostridium bolteae* | 0.007 | 0.024 | 260.387 | 0.128 | 1792.535 | 0.075 | 1001.216 | 0.056 | 732.403 |
| *Lachnoclostridium clostridium fimetarium* | 0.002 | 0.002 | 3.884 | 0.550 | 23276.035 | 0.065 | 2652.876 | 0.007 | 181.980 |
| *Lachnoclostridium clostridium hathewayi* | 0.213 | 0.192 | -10.019 | 0.581 | 172.396 | 0.219 | 2.884 | 0.424 | 98.632 |
| *Lachnoclostridium clostridium hylemonae* | 0.025 | 0.022 | -14.688 | 0.102 | 306.106 | 0.042 | 67.096 | 0.070 | 176.214 |
| *Lachnoclostridium clostridium indolis* | 0.422 | 1.308 | 209.757 | 0.559 | 32.481 | 0.851 | 101.538 | 1.126 | 166.668 |
| *Lachnoclostridium clostridium jejuense* | 0.011 | 0.042 | 281.595 | 0.055 | 408.901 | 0.053 | 386.735 | 0.039 | 262.319 |
| *lachnoclostridium clostridium lavalense* | 0.390 | 0.615 | 57.542 | 1.933 | 395.001 | 1.211 | 210.098 | 0.937 | 140.045 |
| *Lachnoclostridium clostridium oroticum* | 0.001 | 0.001 | -10.883 | 0.002 | 104.103 | 0.002 | 44.472 | 0.001 | 23.175 |
| *Lachnoclostridium clostridium phytofermentans* | 0.013 | 0.010 | -22.276 | 0.005 | -63.781 | 0.007 | -43.206 | 0.012 | -9.105 |
| *Lachnoclostridium clostridium polysaccharolyticum* | 0.017 | 0.052 | 212.070 | 0.044 | 164.261 | 0.059 | 248.671 | 0.169 | 903.148 |
| *Lachnoclostridium clostridium saccharolyticum* | 0.432 | 0.629 | 45.527 | 1.495 | 246.047 | 2.043 | 372.954 | 1.590 | 268.219 |
| *Lachnoclostridium clostridium sphenoides* | 0.052 | 0.219 | 321.803 | 0.212 | 307.072 | 0.092 | 76.112 | 0.176 | 239.119 |
| *Lachnoclostridium clostridium symbiosum* | 0.008 | 0.012 | 63.788 | 0.009 | 16.120 | 0.014 | 88.270 | 0.018 | 146.489 |
| *Lachnoclostridium clostridium xylanolyticum* | 0.725 | 1.478 | 104.027 | 1.225 | 69.029 | 1.110 | 53.200 | 1.873 | 158.489 |
| *lachnoclostridium eubacterium contortum* | 0.005 | 0.002 | -53.599 | 0.004 | -19.627 | 0.023 | 331.443 | 0.012 | 122.590 |
| *Lactobacillus gasseri* | 0.0009 | 0.0011 | 12.171 | 0.001 | 20.273 | 0.001 | 44.712 | 0.0006 | -39.668 |
| *Lactobacillus intestinalis* | 0.001 | 0.000 | -50.991 | 0.003 | 527.494 | 0.004 | 704.991 | 0.00001 | -100.000 |
| *Lactobacillus johnsonii* | 0.074 | 0.056 | -23.744 | 0.103 | 39.663 | 0.087 | 18.890 | 0.068 | -7.645 |
| *Lactobacillus reuteri* | 0.003 | 0.005 | 112.804 | 0.009 | 240.670 | 0.005 | 87.891 | 0.003 | 22.715 |
| *Lactococcus lactis* | 0.025 | 0.015 | -41.873 | 0.025 | 1.217 | 0.026 | 2.500 | 0.012 | -50.496 |
| *Peptoclostridium clostridium hiranonis* | 0.000 | 0.000 | -100.000 | 0.000 | -8.660 | 0.014 | 4879.372 | 0.011 | 3940.688 |
| *Peptoclostridium difficile* | 0.002 | 0.005 | 157.152 | 0.014 | 664.296 | 0.022 | 1143.120 | 0.002 | -1.759 |
| *prevotella spp.* | 0.544 | 0.591 | 8.751 | 3.031 | 457.639 | 3.204 | 489.393 | 0.675 | 24.166 |
| *Ruminiclostridium clostridium alkalicellum* | 0.002 | 0.008 | 205.890 | 0.037 | 1371.696 | 0.017 | 599.785 | 0.016 | 538.800 |
| *Ruminiclostridium clostridium cellobioparum* | 0.000 | 0.002 | 216.389 | 0.001 | 103.874 | 0.001 | 171.820 | 0.003 | 427.368 |
| *Ruminiclostridium clostridium methylpentosum* | 0.025 | 0.100 | 301.733 | 0.117 | 370.434 | 0.120 | 383.967 | 0.092 | 269.656 |
| *Ruminiclostridium clostridium straminisolvens* | 0.006 | 0.008 | 25.097 | 0.030 | 364.156 | 0.012 | 92.891 | 0.014 | 114.662 |
| *Ruminiclostridium clostridium thermocellum* | 0.002 | 0.002 | 15.057 | 0.004 | 72.561 | 0.005 | 121.865 | 0.005 | 136.808 |
| *Ruminiclostridium eubacterium siraeum* | 0.055 | 0.133 | 144.496 | 0.074 | 36.268 | 0.414 | 658.761 | 0.141 | 158.992 |
| *Tyzzerella clostridium colinum* | 0.047 | 0.081 | 71.139 | 0.569 | 1098.326 | 0.314 | 561.409 | 0.141 | 196.985 |
| *Tyzzerella clostridium lactatifermentans* | 0.035 | 0.081 | 131.854 | 0.037 | 6.871 | 0.033 | -4.829 | 0.079 | 127.436 |
| *Tyzzerella clostridium propionicum* | 0.012 | 0.007 | -39.127 | 0.020 | 70.138 | 0.019 | 61.780 | 0.008 | -32.002 |
| **Commulative sum** | **14.851** | **19.852** |  | **25.802** |  | **29.880** |  | **25.093** |  |

**Table S3. List of the LAP bacteria species.**

| Species | Ctrl | PEO | PA | PO | β-PAE |
| --- | --- | --- | --- | --- | --- |
| *Lactobacillus johnsonii* | 0.071±0.15 | 0.038±0.004 | 0.129±0.05 | 0.12±0.1 | 0.07±0.02 |
| *Lactobacillus reuteri* | 0.003±0.002 | 0.005±0.003 | 0.008±0.003 | 0.004±0.003 | 0.003±0.002 |
| *Lactobacillus intestinalis* | 0.001±0.001 | 0.001±0.001 | 0.003±0.002 | 0.006±0.001 | 0.00±0.00 |
| *Lactococcus lactis* | 0.015±0.01 | 0.015±0.01 | 0.034±0.04 | 0.016±0.001 | 0.014±0.003 |
| *Bifidobacterium longum* | 0.002±0.00 | 0.005±0.006 | 0.004±0.00 | 0.005±0.001 | 0.017±0.003 |
| *Faecalibacterium prausnitzii* | 0.001±0.001 | 0.001±0.008 | 0.002±0.00 | 0.005±0.001 | 0.002±0.00 |

**Table S4. List of the primers using in this research.**

| **Target gene** | **Nucleotide sequence of primer (5’ to 3’ )** | |
| --- | --- | --- |
|  | **Forward** | **Reverse** |
| Arginase 1 | TGGCTTGCGAGACGTAGAC | GCTCAGGTGAATCGGCCTTTT |
| E-cadherin | GGACTACGATTATCTGAACG | AACACACACACTATCCAGC |
| CXCL 10 | CCAAGTGCTGCCGTCATTTTC | GGCTCGCAGGGATGATTTCAA |
| β-actin | TGTTACCAACTGGGACGACA | CTGGGTCATCTTTTCACGGT |
| IL-1β | GCTGAAAGCTCTCCACCTCA | GGCCACAGGTATTTTGTCGT |
| FOXP3 | CCCATCCCCAGGAGTCTTG | ACCATGACTAGGGGCACTGTA |
| GPR41 | GGGGTCGATACAAGAGT | CTGGCGGAGCTACGTGCT |
| GPR43 | TTCTTACTGGGCTCCCTGCC | TACCAGCGGAAGTTGGATGC |
| GPR109a | TCAGATCTGACTCGTCCACC | CCATTGCCCAGGAGTCCGAAC |
| MR | GCTGAATCCCAGAAATTCCGC | ATCACAGGCATACAGGGTGAC |
| N-cadherin | GCGAGCCAGCAGATTTCAAG | TTGCCTGCTCTGCAGTGAGA |
| iNOS | GTTCTCAGCCCAACAATACAAGA | GTGGACGGGTCGATGTCAC |
| IL-4 | GGTCTCAACCCCCAGCTAGT | GCCGATGATCTCTCTCAAGTGAT |
| IL-10 | GCTCTTACTGACTGGCATGAG | CGCAGCTCTAGGAGCATGTG |
| IL-6 | CTTCCATCCAGTTGCCTTCTTG | AATTAAGCCTCCGACTTGTGAAG |
| IL-12 | ACTCTGCGCCAGAAACCTC | CACCCTGTTGATGGTCACGAC |
| TNF-α | CAAATGGCCTCCCTCTCAT | CTCCTCCACTTGGTGGTTTG |
| ZO-1 | AATGAATGATGGTTGGTATGG | TGACAGGTAGGACAGACG |
| VCAM-1 | GAACCCAAACAGAGGCAGAG | GGTATCCCATCACTTGAGCAG |
| ICAM-1 | CGCTGTGCTTTGAGAACTGT | AGGTCCTTGCCTACTTGCTG |
| ERIC | ATGTAAGCTCCTGGGGATTCAC | AAGTAAGTGACTGGGGTGAGCG |
| PPAR-ɤ | CTGCTCAAGTATGGTGTCCATGA | TGAGATGAGGACTCCATCTTTATTCA |
| Occludin | ATGGCAAGCGATCATACCC | TTCCTGCTTTCCCCTTCG |
|  |  |  |
